# Supplementary material for: Antibacterial Activities and Life Cycle Stages of Asparagopsis armata: Implications of the Metabolome and Microbiome
Source: Mar Drugs. 2023 Jun 17;21(6):363. doi: 10.3390/md21060363 (PMC10301895; doi:10.3390/md21060363)

Supplementary materials

Antibacterial Activities and Life Cycle Stages of *Asparagopsis armata*: Implications of the Metabolome and Microbiome

Christelle Parchemin*[,1,2], Delphine Raviglione[1], Anouar Mejait[1], Pierre Sasal[1], Elisabeth Faliex[2], Camille Clerissi[1], Nathalie Tapissier-Bontemps[1]

[1] **C**entre de **R**echerches **I**nsulaires et **Ob**servatoire de l'**E**nvironnement (CRIOBE), UAR 3278 UPVD-EPHE-CNRS, Université de Perpignan - Via Domitia, 52 Av. Paul Alduy, 66860 Perpignan CEDEX, France

[2] **CE**ntre de **F**ormation et de **R**echerche sur les **E**nvironnements **M**éditerranéens (CEFREM), UMR 5110 UPVD-CNRS, Université de Perpignan - Via Domitia, 52 Av. Paul Alduy, 66860 Perpignan CEDEX, France

[*]Corresponding authors: Christelle Parchemin (christelle.parchemin@univ-perp.fr); Nathalie Tapissier-Bontemps (nathalie.tapissier@univ-perp.fr)

Table of contents

Supplementary methods 4

Supplementary method 1: Pictures of the stages of *A. armata* life cycle collected in Banyuls-sur-Mer with the tetrasporophyte (A), the gametophyte without cystocarps (B) and the gametophyte with developed cystocarps (C)... 4

Supplementary method 2: Details of data pre-processing for the metabolomics study............................ 4

Supplementary method 3: Composition of bacterial media. The quantities (in g) correspond to the preparation of 1 L of medium.. 5

Supplementary figures... 6

Figure S1: Hierarchical clustering analysis of the metabolome of three stages of the life cycle of *A. armata* analysed in UHPLC-ESI(-)-HRMS/MS (distance measure: Euclidean, clustering algorithm: Ward)...... 6

Figure S2: Chromatogram of a quality control sample analysed with the UHPLC-ESI(-)-HRMS spectrometer and displaying the ion at *m/z* 572.50134 $[M\text{-}H]^-$ (most intense isotopic mass) of the molecule $C_5H_2Br_6O_2$ with the highest intensity on the chromatogram. 7

Figure S3: Chromatogram of the most active fractions (flash-chromatography fractionation) analysed by UHPLC-ESI(-)-HRMS/MS 8

Figure S4: ESI(-)-MS/MS spectrum of $C_5H_2Br_6O_2$ (compound 1) and the possible fragmentation pattern. 9

Figure S5: ESI(-)-MS/MS spectrum of $C_5H_2Br_5ClO_2$ (compound 2) and the possible fragmentation pattern... 9

Figure S6: ESI(-)-MS/MS spectrum of $C_5H_3Br_4ClO_2$ (compound 5) and the possible fragmentation pattern... 10

Figure S7: ESI(-)-MS/MS spectrum of $C_4H_3Br_3O_3$ (compound 6) and the possible fragmentation pattern. 10

Figure S8: ESI(-)-MS/MS spectrum of $C_5H_3Br_5O_2$ (compound 7) and the possible fragmentation pattern. 11

Figure S9: Chromatogram of the most active fractions of the second fractionation (HPLC (Waters 1525) coupled to a UV detector (Waters 2487)) analysed by UHPLC-ESI(-)-HRMS/MS 12

Figure S10: EIC of ion with a *m/z* 572.5021 (most abundant isotopic mass) corresponding to a monoisotopic m/z of 566.5085 in active fractions, including 40 % H_2O-MeOH (A), 30 % H_2O-MeOH (B), F9 (C) and F10 (D)... 13

Figure S11: Rarefaction curves of 16S rRNA gene sequences for the gametophyte with developed cystocarps (A), the gametophyte (B) and the tetrasporophyte (C) samples. 14

Figure S12: Relative abundance of bacterial families associated with the three *A. armata* stages. Codes "C" represents samples of the gametophyte with developed cystocarps, "G" the gametophyte samples and "T" the tetrasporophyte samples. 15

Figure S13: KO pathways of the most predicted abundant functions associated with GC (A), G (B) and T (C).. 16

Figure S14: Scores plot of the multiblock PLS-DA analysis (DIABLO) of *A. armata* gametophyte *(G)*, with developed cystocarps *(GC)*, and tetrasporophyte *(T)* phases. 17

Figure S15: Correlations between the first dimension (A) and the second dimension (B) of each dataset (Chem= Chemistry; Metabarc= Metabarcoding) for the two PLS models. Codes "C" represents samples of the gametophyte with developed cystocarps, "G" the gametophyte samples and "T" the tetrasporophyte samples. 18

Figure S16: Heatmap of significant ASV and metabolites of the three phases of *A. armata* ("C" represents samples of the gametophyte with developed cystocarps, "G" the gametophyte samples and "T" the tetrasporophyte samples)... 19

Supplementary methods

Supplementary method 1: Pictures of the stages of *A. armata* life cycle collected in Banyuls-sur-Mer with the tetrasporophyte (A), the gametophyte without cystocarps (B) and the gametophyte with developed cystocarps (C)

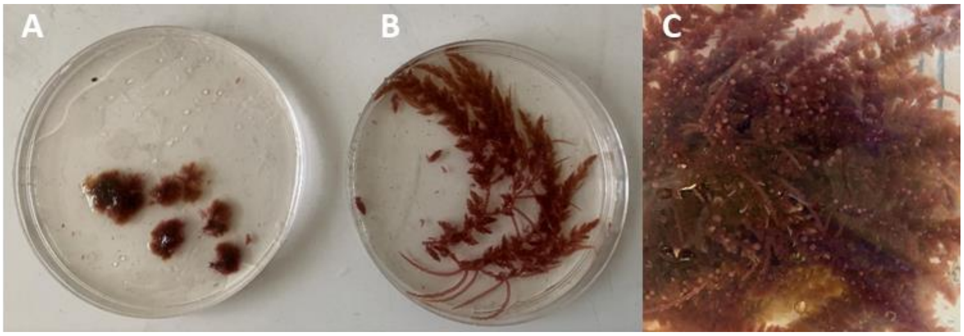

Supplementary method 2: Details of data pre-processing for the metabolomics study

Data acquisitions were performed using Xcalibur 4.1.31.9 (Thermo Fisher Scientific). Raw data were converted to mzML files with MSconvert (version 3.0, from Proteowizard library). mzML files were uploaded and processed using the Galaxy web platform (version 3.3) (Giacomoni et al., 2015; Guitton et al., 2017). The workflow used for data pre-processing and used parameters are published on the Galaxy Workflow4Metabolomics platform at: https://workflow4metabolomics.usegalaxy.fr/u/christelle_parchemin/w/workflowparcheminalgae. Briefly, the preprocessing consisted in a chromatographic peak detection (Galaxy Version 3.12+galaxy0) using the CentWave method with a minimum and maximum peak width of 5 and 60s, and 4 successive scans with an intensity above 500 000 as limit for consideration of region of interest. The chromotagraphic peak detection was followed by a peak grouping using the PeakDensity method step, a loess/non-linear “PeakGroups” retention time adjustment (degree of smoothing: 0.8), a peak filling and “CAMERA” peak annotation. A matrix of features with peak intensity, m/z value and retention time was generated. A clean-up based on p-Values and t-Stat outputs generated by the “CAMERA” step was performed in order to eliminate all features that are significantly detected in blanks. Then, an “inter/intra-batch” signal correction was applied using the “Batch correction” function with a “loess” regression model (span = 0.8) (Van Der Kloet et al., 2009). This step was followed by a second clean-up according to feature's CV in pool QC injections (all features with area relative standard deviation upper than 30% through pool QC injections were eliminated from the

dataset) (Thévenot et al., 2015). Finally, redundancies due to isotopes were manually eliminated (only monoisotopic mass was kept). For identification, most probable molecular formula was determined using using Sirius (v4.9.15 (Dührkop et al., 2019))), MetLin database, characteristics isotopic clusters, MS/MS spectra and comparison with literature.

Supplementary method 3: Composition of bacterial media. The quantities (in g) correspond to the preparation of 1 L of medium

Code	Strain	Medium	Culture temperature (°C)	Incubation time (h)	Susceptible to antibiotic
Ea	*Edwardsiella anguillarum* (DSMZ-27202)	Luria Broth[a]	27	24	Kanamycine
Lg	*Lactococcus garvieae* (CIP102507T)	Brain Heart Infusion[b]	37	24	Erythromycine
Tm	*Tenacibaculum martimum* (CIP103528T)	Marine Broth[c]	27	24	Ampicilline
Va	*Vibrio anquillarum* (CIP 63.36T)	Marine Broth[c]	27	24	Kanamycine
Vh	*Vibrio harveyi* (CIP103192T)	Marine Broth[c]	27	24	Kanamycine
Yr	*Yersina ruckeri* (CIP82.80T)	Marine Broth[c]	27	24	Amoxicilline

[a]Luria Broth = 5 g of yeast extract (Yeast Extract 70161-500G (Fluka Analytical, Sigma-Aldrich®, Saint-Louis USA)), 10g of peptone (Peptone from casein, pancreatic digest 70169-500G (Fluka Analytical, Sigma-Aldrich®, Saint-Louis USA)), 10g of sea salts (Sea salts S9883-500G (Sigma Life Science, Sigma-Aldrich®, Saint-Louis USA)) and 15 g of bacteriological agar (Bacteriological agar A5306-250G (Sigma-Aldrich®, Saint-Louis USA)).
[b]Brain Heart Infusion Agar = 52 g for 1 L of medium (Brain Heart Infusion Agar 70138-500G (Sigma-Aldrich®, Saint-Louis USA))
[c]Marine Broth = 37.4 g for 1 L of medium (Difco™ Marine Broth 2216 (Becton, Dickinson and Company, Sparks, USA))

<u>Supplementary figures</u>

Figure S1: Hierarchical clustering analysis of the metabolome of three stages of the life cycle of *A. armata* analysed in UHPLC-ESI(−)-HRMS/MS (distance measure: Euclidean, clustering algorithm: Ward)

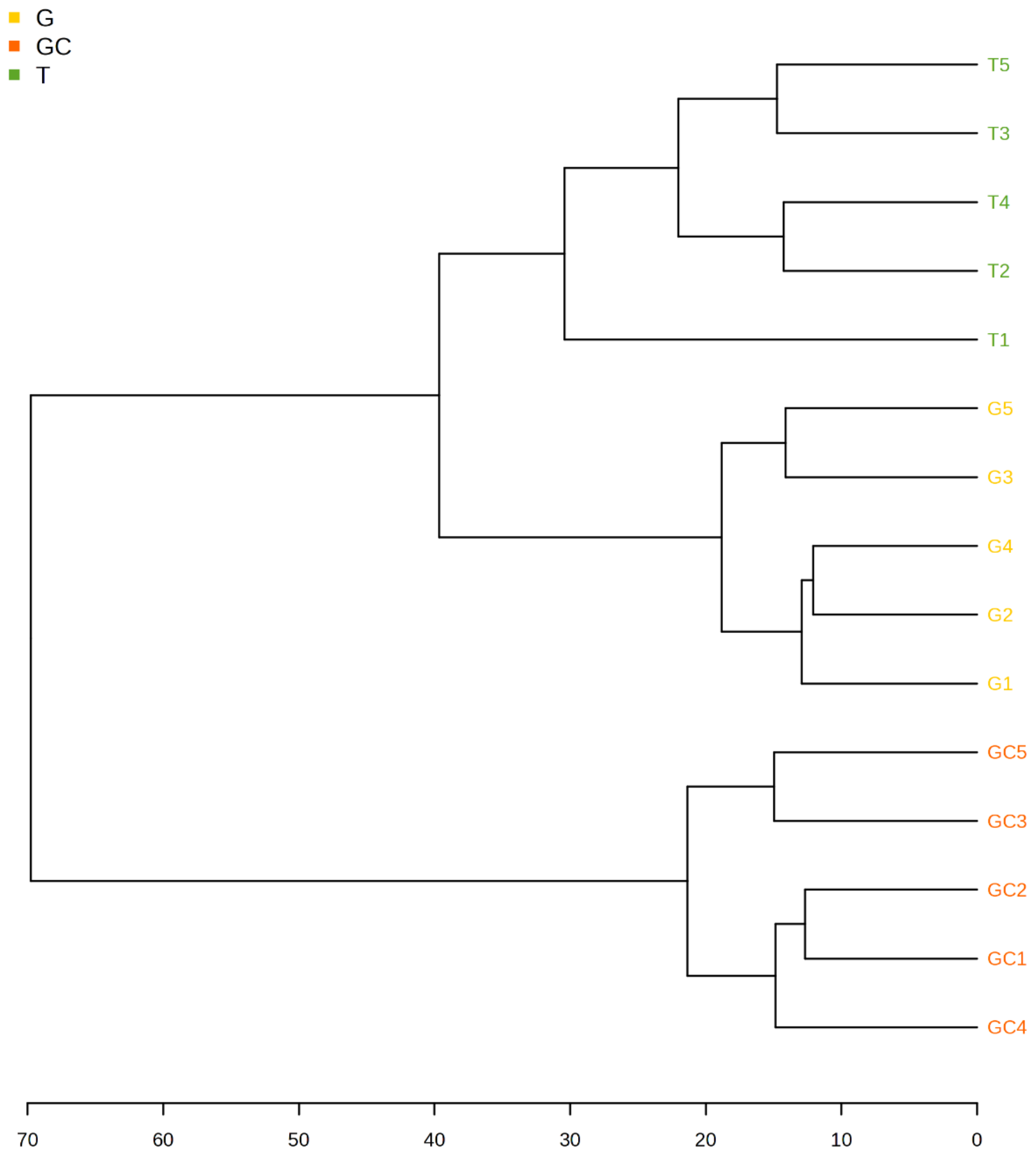

Figure S2: Chromatogram of a quality control sample analysed with the UHPLC-ESI(−)-HRMS spectrometer and displaying the ion at *m/z* 572.50134 [M−H]$^-$ (most intense isotopic mass) of the molecule $C_5H_2Br_6O_2$ with the highest intensity on the chromatogram.

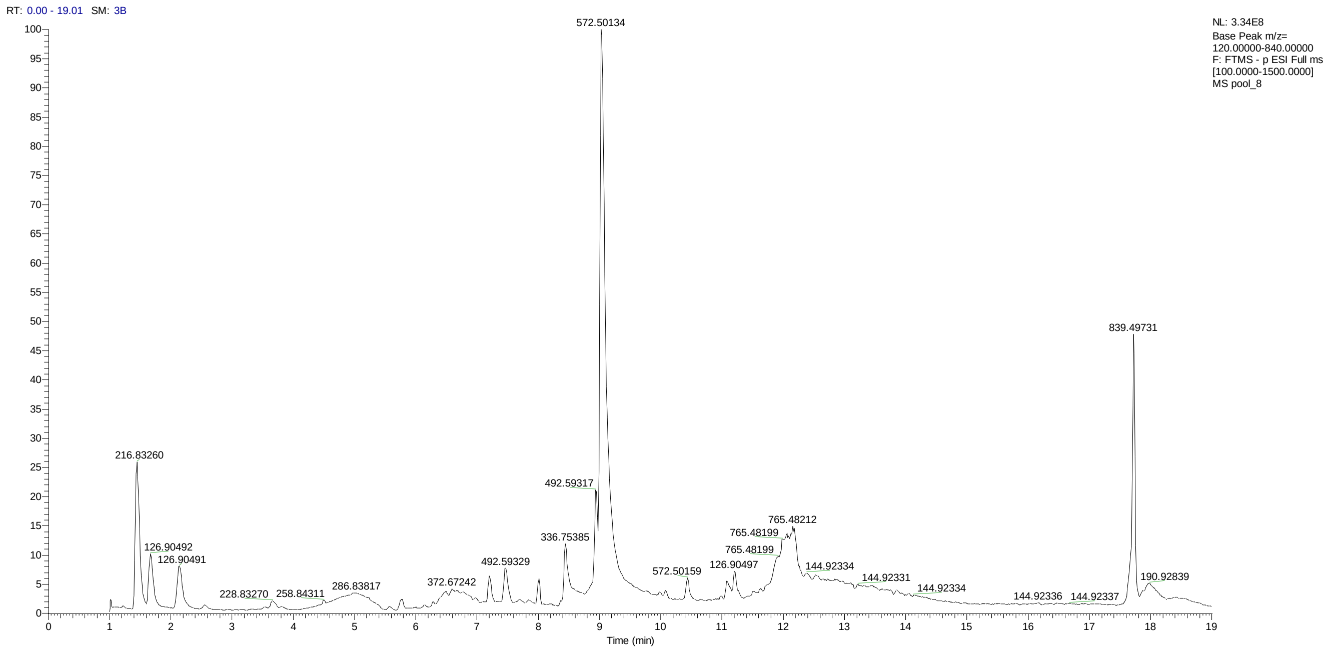

Figure S3: Chromatogram of the most active fractions (flash-chromatography fractionation) analysed by UHPLC-ESI(−)-HRMS/MS

A. 40 % H_2O-MeOH (98.9±0.4 % of bacterial growth inhibition)

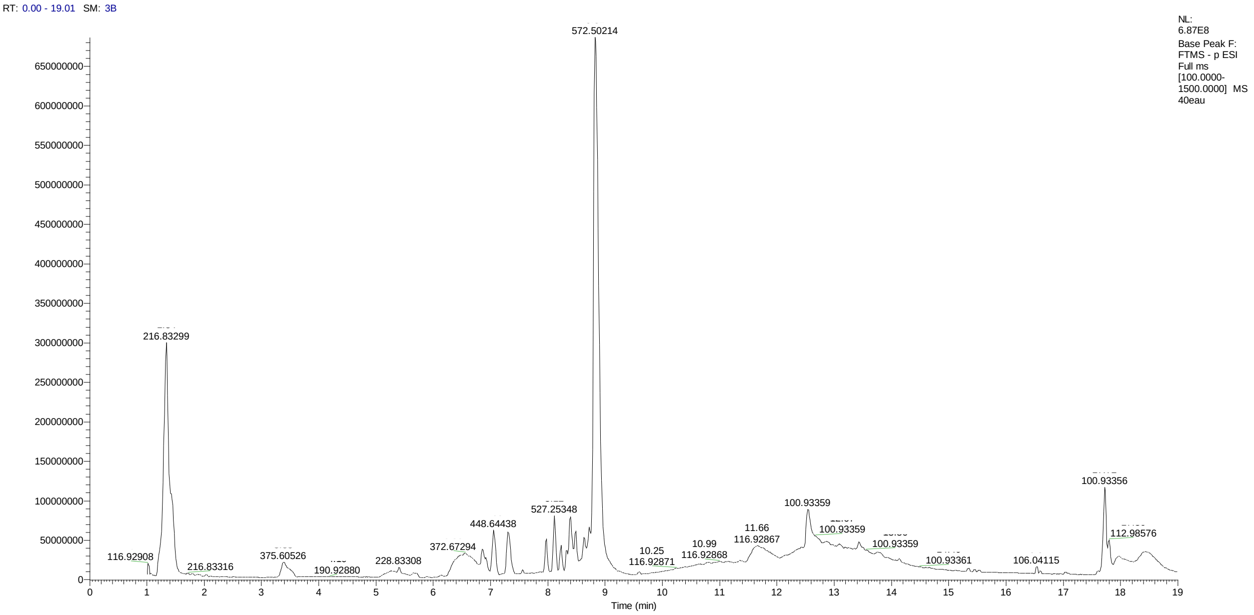

B. 30 % H_2O-MeOH (97.6±0.8 % of bacterial growth inhibition)

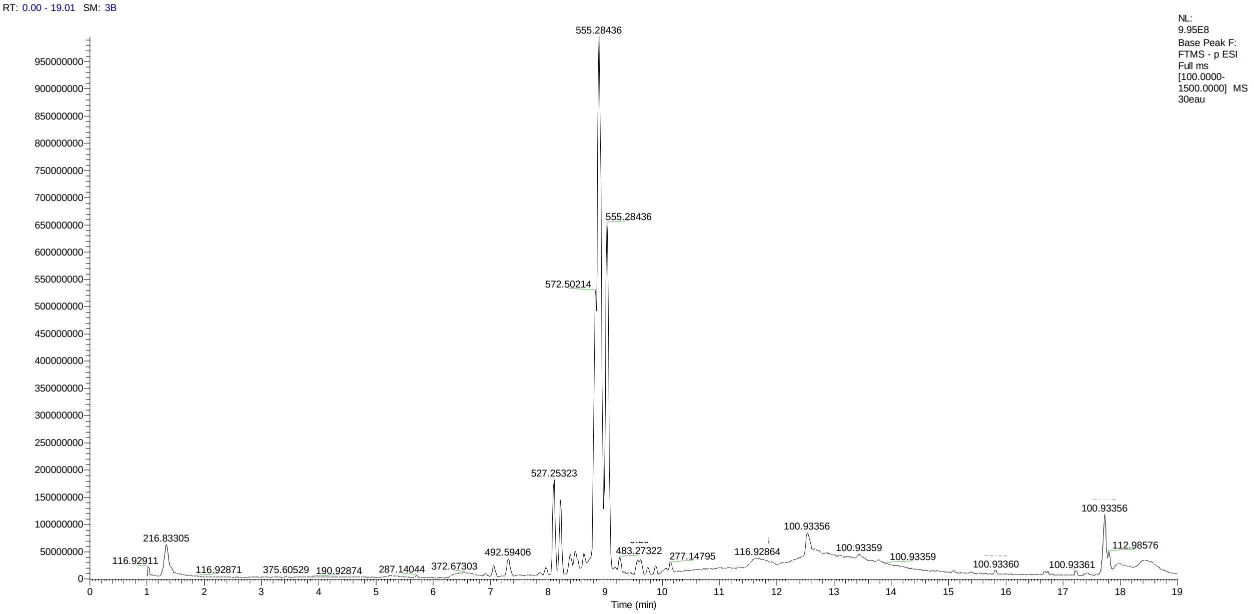

Figure S4: ESI(−)-MS/MS spectrum of $C_5H_2Br_6O_2$ (compound 1) and the possible fragmentation pattern.

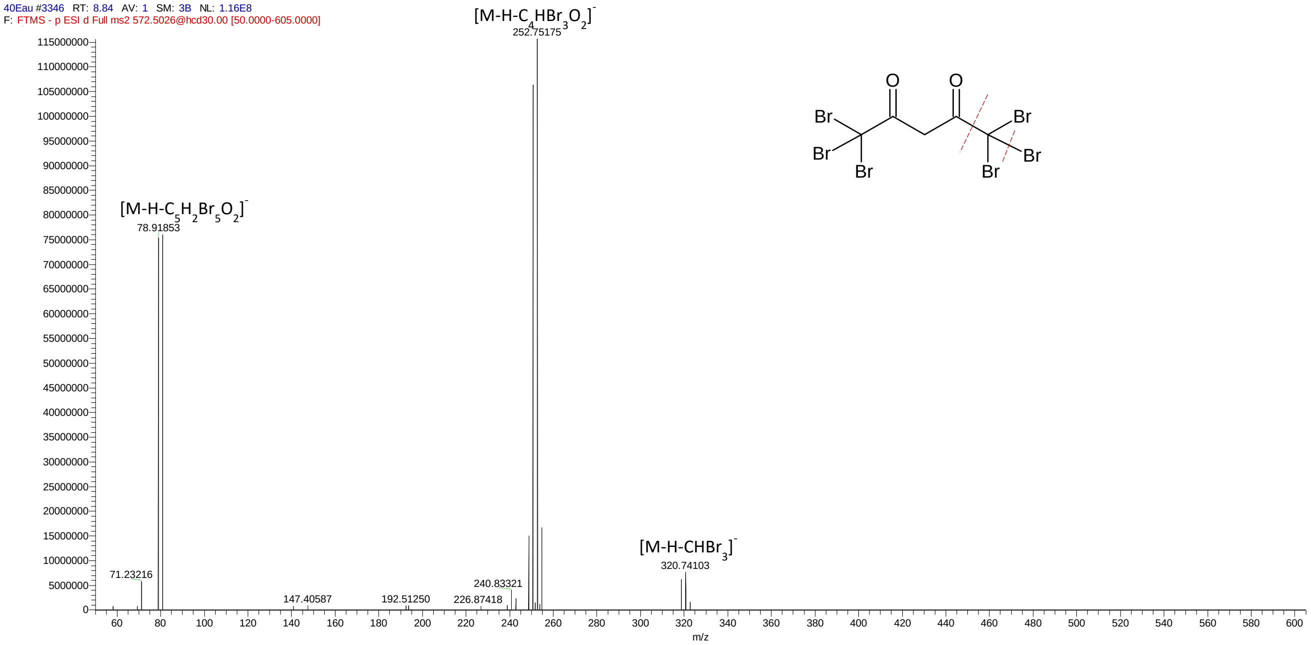

Figure S5: ESI(−)-MS/MS spectrum of $C_5H_2Br_5ClO_2$ (compound 2) and the possible fragmentation pattern.

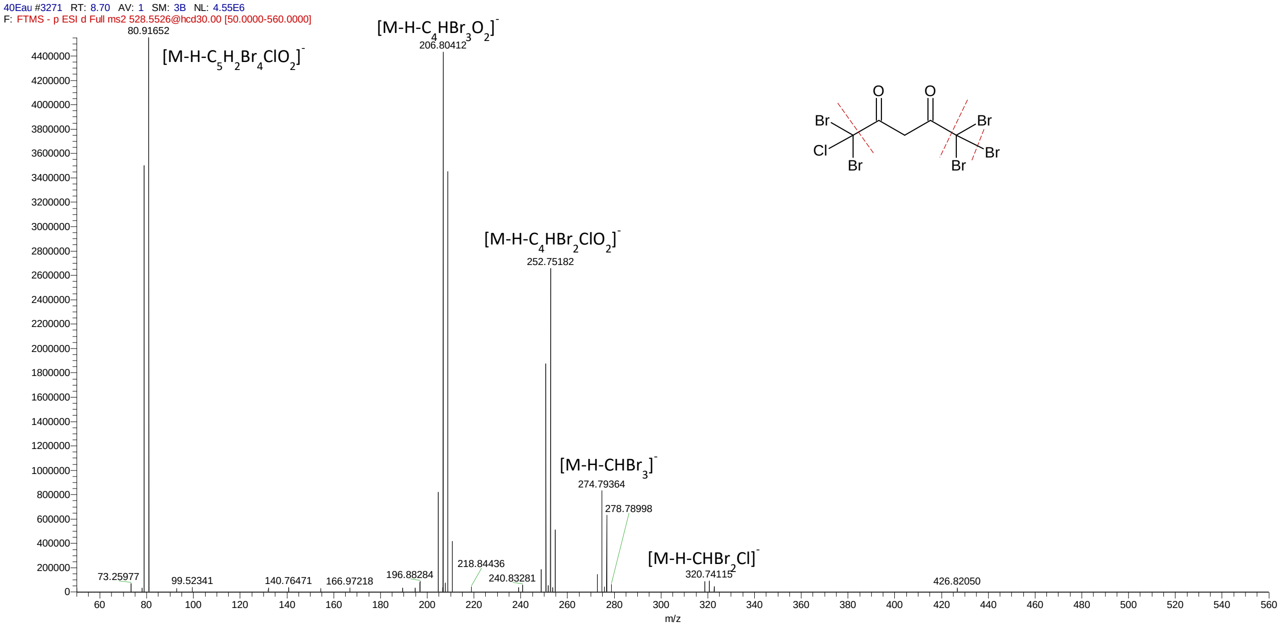

Figure S6: ESI(−)-MS/MS spectrum of $C_5H_3Br_4ClO_2$ (compound 5) and the possible fragmentation pattern.

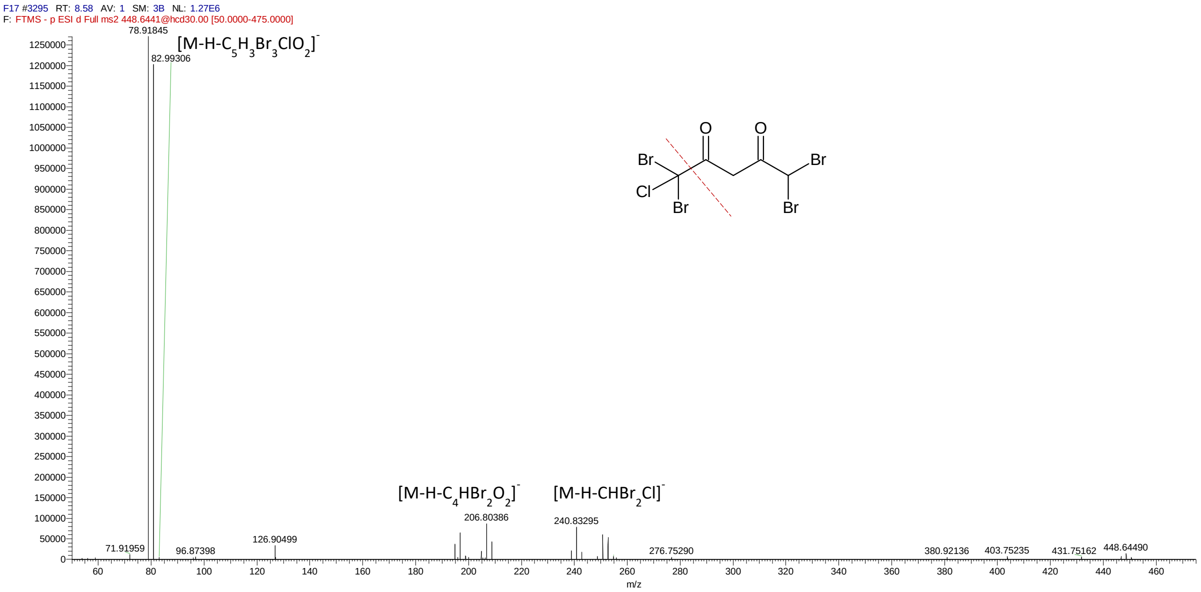

Figure S7: ESI(−)-MS/MS spectrum of $C_4H_3Br_3O_3$ (compound 6) and the possible fragmentation pattern.

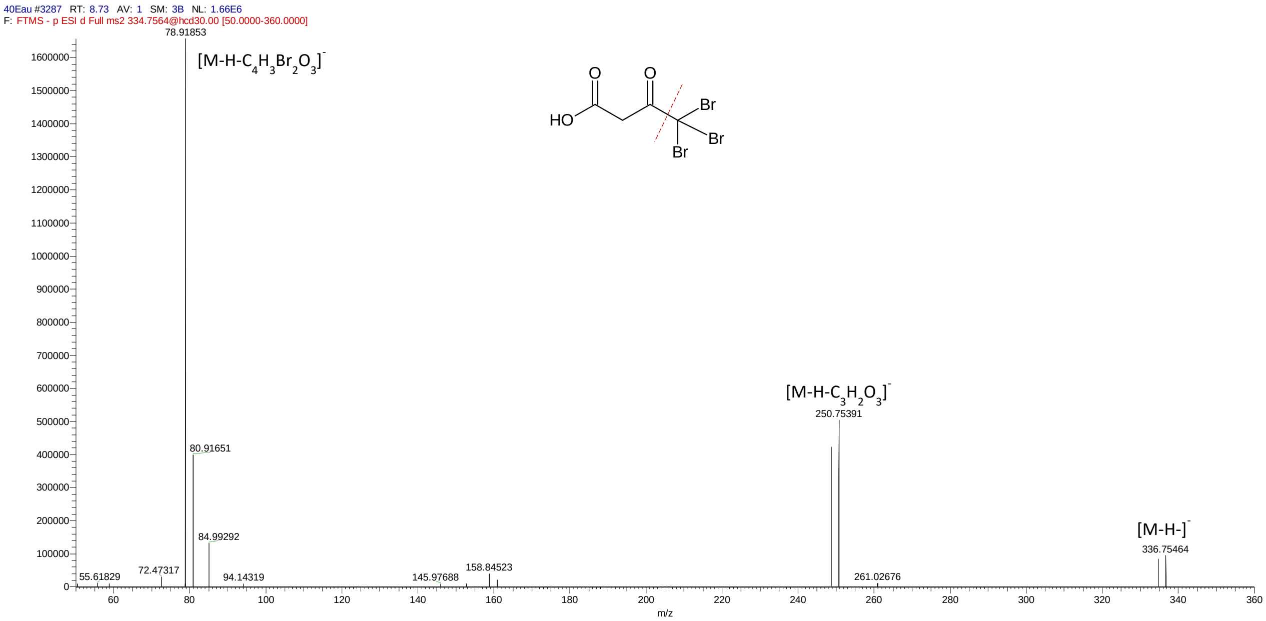

Figure S8: ESI(−)-MS/MS spectrum of $C_5H_3Br_5O_2$ (compound 7) and the possible fragmentation pattern.

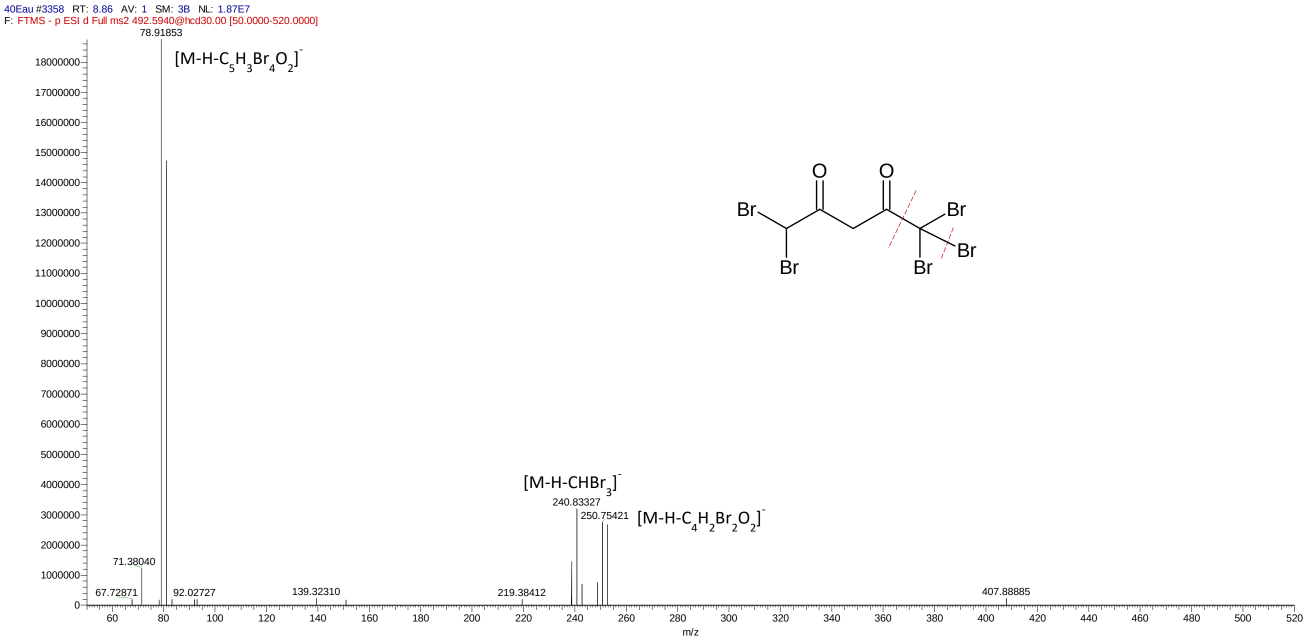

Figure S9: Chromatogram of the most active fractions of the second fractionation (HPLC (Waters 1525) coupled to a UV detector (Waters 2487)) analysed by UHPLC-ESI(−)-HRMS/MS

A. F9 (99±0.0 % of bacterial growth inhibition)

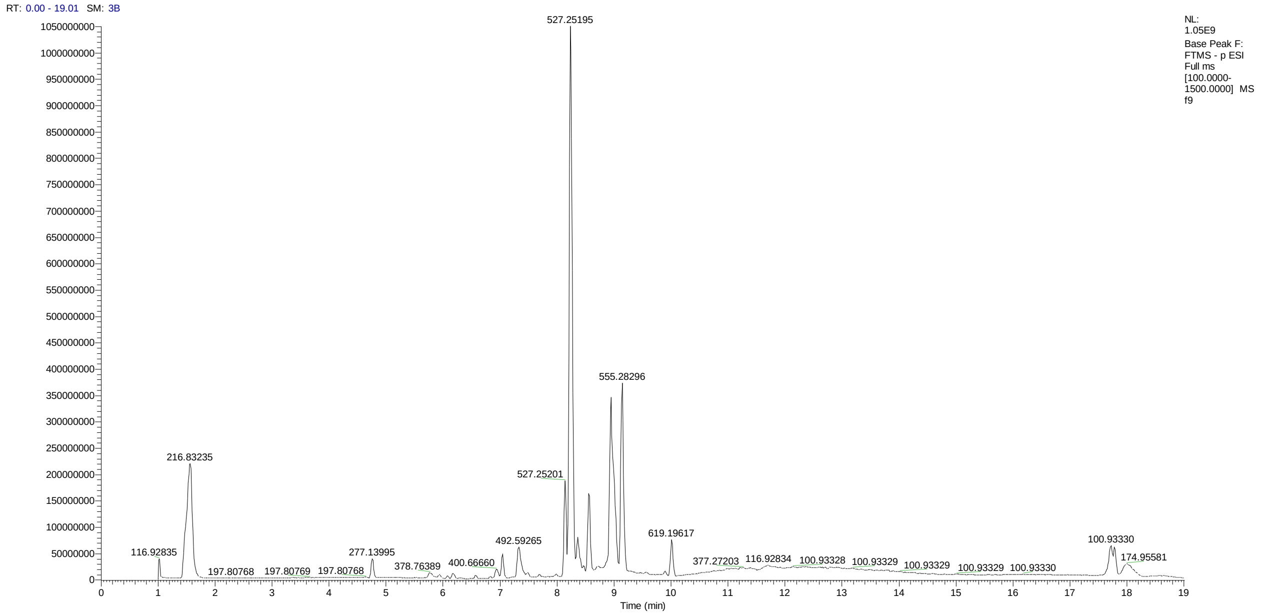

B. F10 (93.7±0.3 % of bacterial growth inhibition)

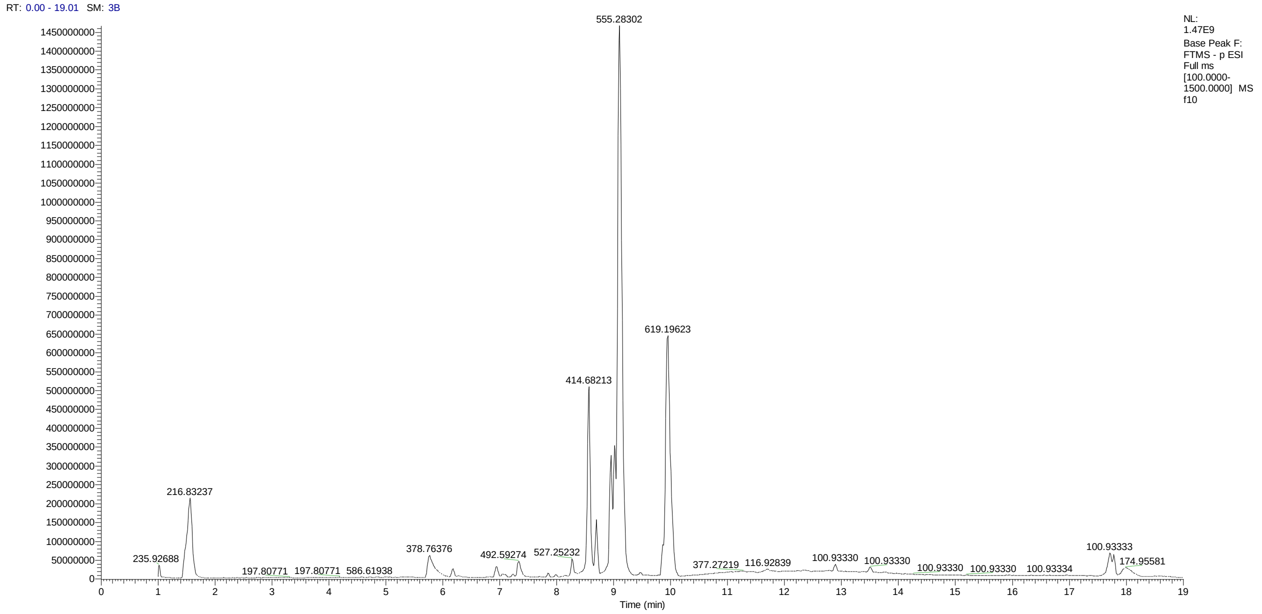

Figure S10: EIC of ion with a *m/z* 572.5021 (most abundant isotopic mass) corresponding to a monoisotopic m/z of 566.5085 in active fractions, including 40 % H_2O-MeOH (A), 30 % H_2O-MeOH (B), F9 (C) and F10 (D).

A. 40% H_2O-MeOH

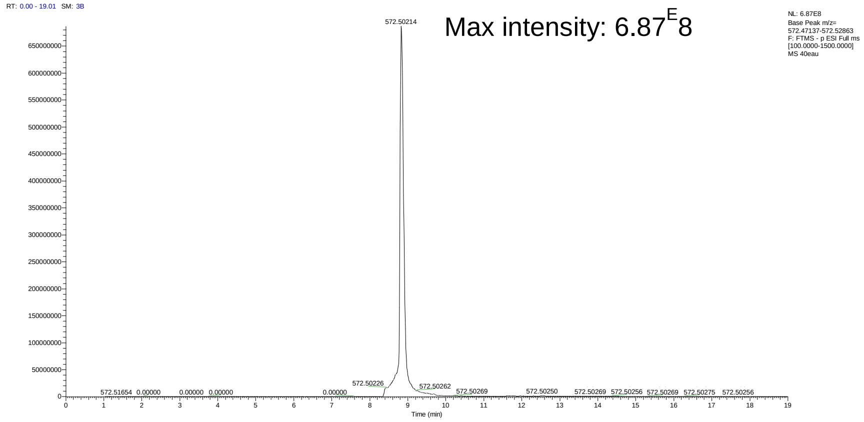

B. 30% H_2O-MeOH

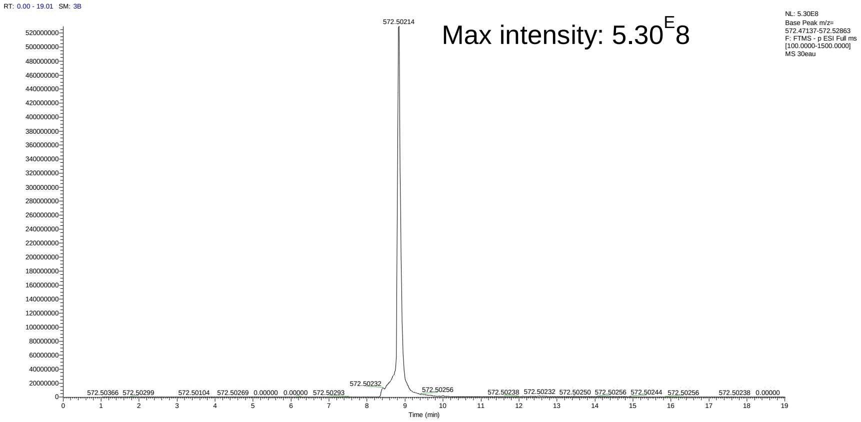

C. F9

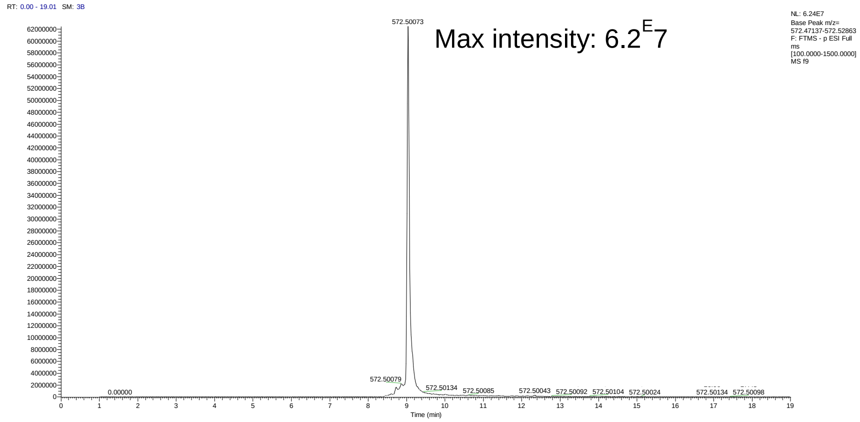

D. F10

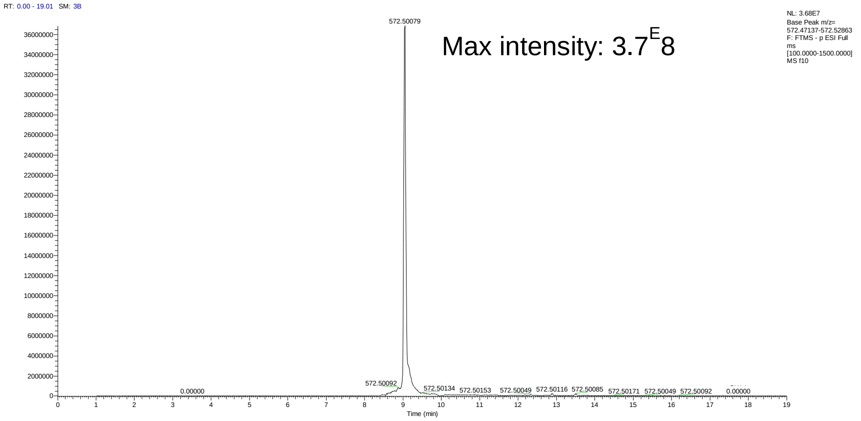

Figure S11: Rarefaction curves of 16S rRNA gene sequences for the gametophyte with developed cystocarps (A), the gametophyte (B) and the tetrasporophyte (C) samples.

A.

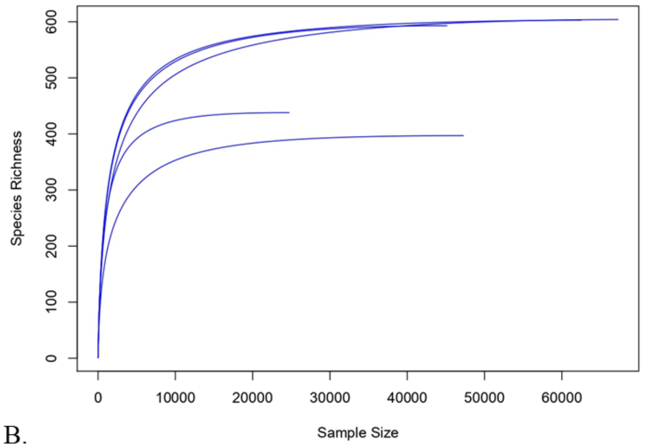

B.

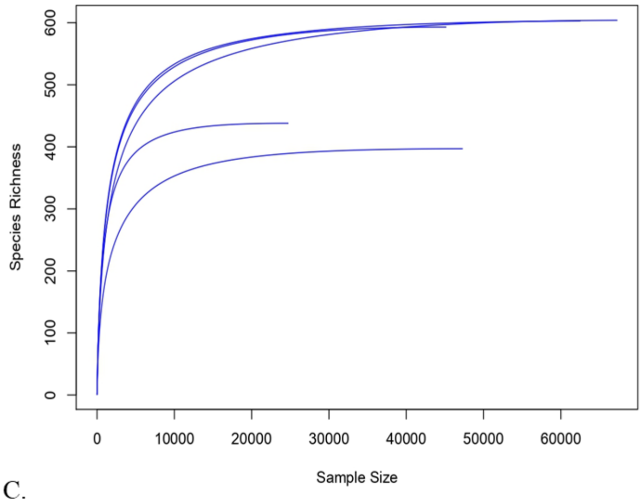

C.

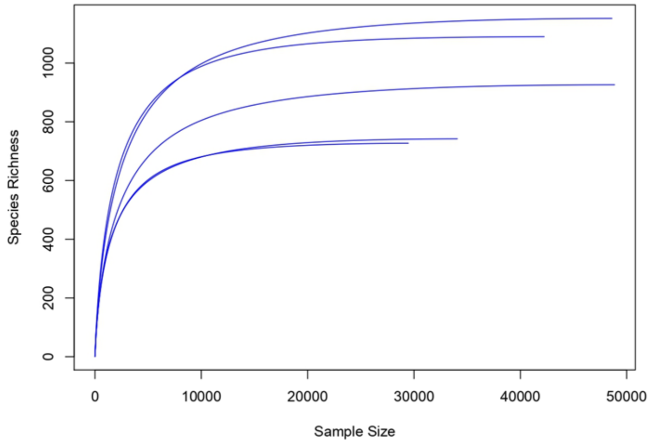

Figure S12: Relative abundance of bacterial families associated with the three *A. armata* stages. Codes "C" represents samples of the gametophyte with developed cystocarps, "G" the gametophyte samples and "T" the tetrasporophyte samples.

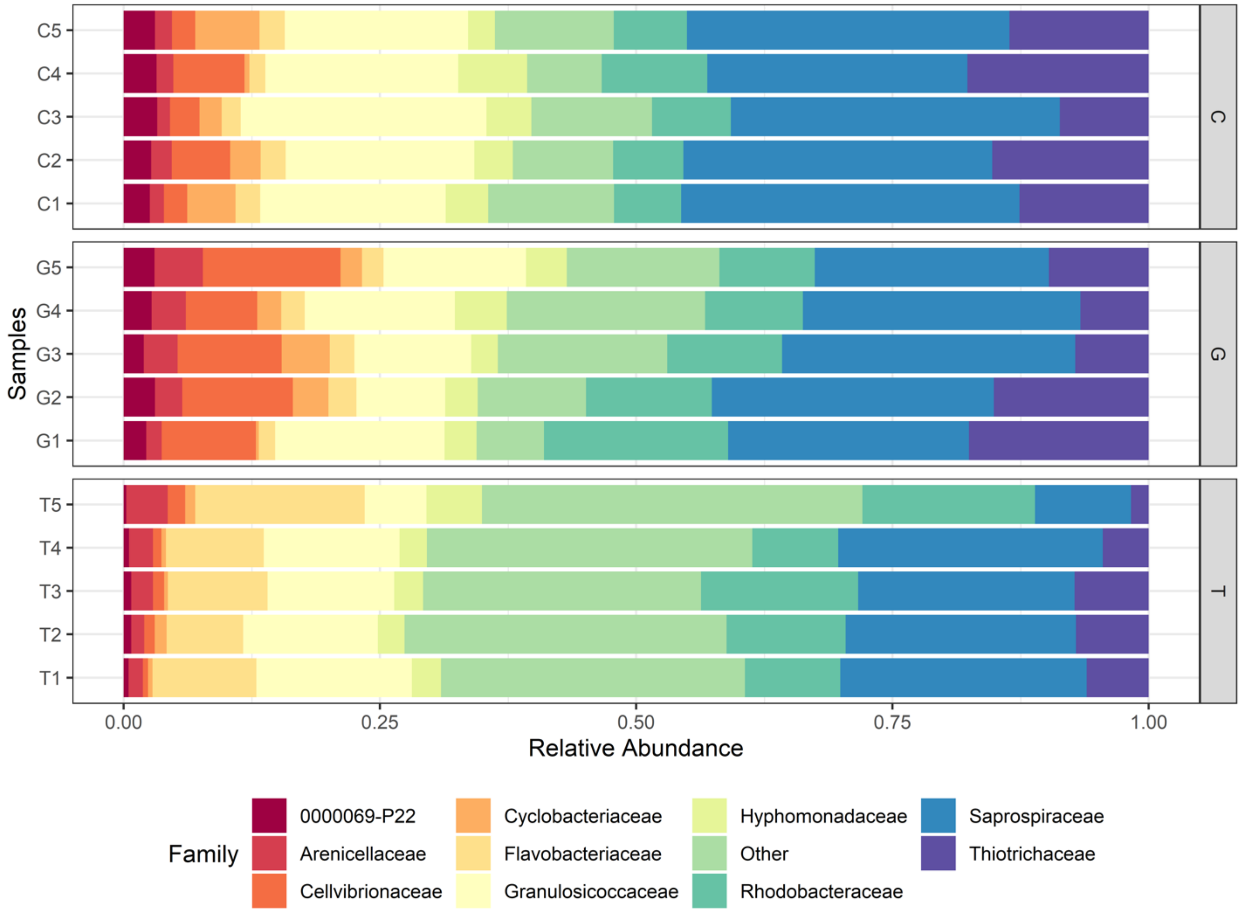

Figure S13: KO pathways of the most predicted abundant functions associated with GC (A), G (B) and T (C).

A. GC

Metabolism

- Global and overview maps
  - 01100 Metabolic pathways (4)
- Carbohydrate metabolism
  - 00040 Pentose and glucuronate interconversions (1)
  - 00650 Butanoate metabolism (1)
- Energy metabolism
  - 00920 Sulfur metabolism (1)
- Nucleotide metabolism
  - 00230 Purine metabolism (1)
- Amino acid metabolism
  - 00260 Glycine, serine and threonine metabolism (1)

Environmental Information Processing

- Membrane transport
  - 02010 ABC transporters (5)
- Signal transduction
  - 02020 Two-component system (2)

Cellular Processes

- Cell growth and death
  - 04113 Meiosis - yeast (1)
- Cellular community - prokaryotes
  - 02024 Quorum sensing (1)
  - 02025 Biofilm formation - Pseudomonas aeruginosa (1)
  - 02026 Biofilm formation - Escherichia coli (1)

Organismal Systems

- Aging
  - 04213 Longevity regulating pathway - multiple species (1)

Human Diseases

- Drug resistance: antimicrobial
  - 01503 Cationic antimicrobial peptide (CAMP) resistance (1)

B. G

Metabolism

- Global and overview maps
  - 01100 Metabolic pathways (25)
  - 01110 Biosynthesis of secondary metabolites (12)
  - 01120 Microbial metabolism in diverse environments (15)
  - 01200 Carbon metabolism (7)
  - 01210 2-Oxocarboxylic acid metabolism (1)
  - 01212 Fatty acid metabolism (8)
  - 01230 Biosynthesis of amino acids (2)
  - 01240 Biosynthesis of cofactors (1)
- Carbohydrate metabolism
  - 00010 Glycolysis / Gluconeogenesis (2)
  - 00053 Ascorbate and aldarate metabolism (1)
  - 00620 Pyruvate metabolism (5)
  - 00630 Glyoxylate and dicarboxylate metabolism (5)
  - 00640 Propanoate metabolism (6)
  - 00650 Butanoate metabolism (6)
  - 00660 C5-Branched dibasic acid metabolism (1)
- Energy metabolism
  - 00720 Carbon fixation pathways in prokaryotes (3)
  - 00680 Methane metabolism (1)
  - 00910 Nitrogen metabolism (1)
  - 00920 Sulfur metabolism (1)
- Lipid metabolism
  - 00061 Fatty acid biosynthesis (3)
  - 00071 Fatty acid degradation (8)
  - 00120 Primary bile acid biosynthesis (1)
  - 00561 Glycerolipid metabolism (1)
  - 00592 alpha-Linolenic acid metabolism (1)
- Amino acid metabolism
  - 00250 Alanine, aspartate and glutamate metabolism (2)
  - 00260 Glycine, serine and threonine metabolism (1)
  - 00280 Valine, leucine and isoleucine degradation (11)
  - 00290 Valine, leucine and isoleucine biosynthesis (1)
  - 00310 Lysine degradation (5)
  - 00220 Arginine biosynthesis (2)
  - 00330 Arginine and proline metabolism (1)
  - 00340 Histidine metabolism (1)
  - 00350 Tyrosine metabolism (1)
  - 00360 Phenylalanine metabolism (2)
  - 00380 Tryptophan metabolism (4)
- Metabolism of other amino acids
  - 00410 beta-Alanine metabolism (3)
  - 00480 Glutathione metabolism (1)
- Metabolism of cofactors and vitamins
  - 00760 Nicotinate and nicotinamide metabolism (1)
  - 00770 Pantothenate and CoA biosynthesis (2)
- Metabolism of terpenoids and polyketides
  - 00900 Terpenoid backbone biosynthesis (1)
  - 00981 Insect hormone biosynthesis (1)
  - 00903 Limonene and pinene degradation (2)
  - 00281 Geraniol degradation (2)
- Xenobiotics biodegradation and metabolism
  - 00362 Benzoate degradation (4)
  - 00627 Aminobenzoate degradation (1)
  - 00625 Chloroalkane and chloroalkene degradation (1)
  - 00642 Ethylbenzene degradation (1)
  - 00791 Atrazine degradation (1)
  - 00930 Caprolactam degradation (2)
  - 00980 Metabolism of xenobiotics by cytochrome P450 (1)
  - 00982 Drug metabolism - cytochrome P450 (1)
  - 00983 Drug metabolism - other enzymes (1)

Genetic Information Processing

- Translation
  - 00970 Aminoacyl-tRNA biosynthesis (1)

Environmental Information Processing

- Membrane transport
  - 02010 ABC transporters (18)
- Signal transduction
  - 02020 Two-component system (3)

Cellular Processes

- Transport and catabolism
  - 04146 Peroxisome (2)
- Cell growth and death
  - 04216 Ferroptosis (1)
  - 04217 Necroptosis (1)
- Cellular community - prokaryotes
  - 02024 Quorum sensing (14)

Organismal Systems

- Endocrine system
  - 04920 Adipocytokine signaling pathway (1)
  - 03320 PPAR signaling pathway (2)
- Digestive system
  - 04975 Fat digestion and absorption (1)
- Nervous system
  - 04724 Glutamatergic synapse (1)
  - 04727 GABAergic synapse (1)
- Aging
  - 04212 Longevity regulating pathway - worm (1)
- Environmental adaptation
  - 04714 Thermogenesis (1)

Human Diseases

- Cancer: overview
  - 05200 Pathways in cancer (1)
  - 05204 Chemical carcinogenesis - DNA adducts (1)
  - 05207 Chemical carcinogenesis - receptor activation (1)
  - 05208 Chemical carcinogenesis - reactive oxygen species (1)
- Cancer: specific types
  - 05225 Hepatocellular carcinoma (1)
- Cardiovascular disease
  - 05418 Fluid shear stress and atherosclerosis (1)
- Endocrine and metabolic disease
  - 04936 Alcoholic liver disease (2)
- Drug resistance: antimicrobial
  - 01501 beta-Lactam resistance (2)
- Drug resistance: antineoplastic
  - 01524 Platinum drug resistance (1)

C. T

Metabolism

- Global and overview maps
  - 01100 Metabolic pathways (6)
  - 01110 Biosynthesis of secondary metabolites (5)
  - 01120 Microbial metabolism in diverse environments (3)
  - 01200 Carbon metabolism (3)
  - 01212 Fatty acid metabolism (1)
  - 01230 Biosynthesis of amino acids (1)
  - 01250 Biosynthesis of nucleotide sugars (1)
  - 01240 Biosynthesis of cofactors (2)
- Carbohydrate metabolism
  - 00010 Glycolysis / Gluconeogenesis (2)
  - 00020 Citrate cycle (TCA cycle) (2)
  - 00052 Galactose metabolism (1)
  - 00520 Amino sugar and nucleotide sugar metabolism (1)
  - 00620 Pyruvate metabolism (2)
  - 00630 Glyoxylate and dicarboxylate metabolism (1)
  - 00640 Propanoate metabolism (1)
  - 00562 Inositol phosphate metabolism (1)
- Energy metabolism
  - 00680 Methane metabolism (1)
- Lipid metabolism
  - 00061 Fatty acid biosynthesis (1)
- Amino acid metabolism
  - 00260 Glycine, serine and threonine metabolism (2)
  - 00270 Cysteine and methionine metabolism (1)
  - 00280 Valine, leucine and isoleucine degradation (1)
  - 00310 Lysine degradation (1)
  - 00380 Tryptophan metabolism (1)
- Glycan biosynthesis and metabolism
  - 00541 O-Antigen nucleotide sugar biosynthesis (1)
- Metabolism of cofactors and vitamins
  - 00780 Biotin metabolism (1)
- Biosynthesis of other secondary metabolites
  - 00521 Streptomycin biosynthesis (1)
  - 00333 Prodigiosin biosynthesis (1)

Environmental Information Processing

- Membrane transport
  - 02010 ABC transporters (3)
- Signal transduction
  - 02020 Two-component system (4)
  - 04070 Phosphatidylinositol signaling system (1)

Cellular Processes

- Cell growth and death
  - 04112 Cell cycle - Caulobacter (2)
- Cellular community - prokaryotes
  - 02025 Biofilm formation - Pseudomonas aeruginosa (1)
- Cell motility
  - 02030 Bacterial chemotaxis (2)
  - 02040 Flagellar assembly (1)

Figure S14: Scores plot of the multiblock PLS-DA analysis (DIABLO) of *A. armata* gametophyte (G), with developed cystocarps (GC), and tetrasporophyte (T) phases.

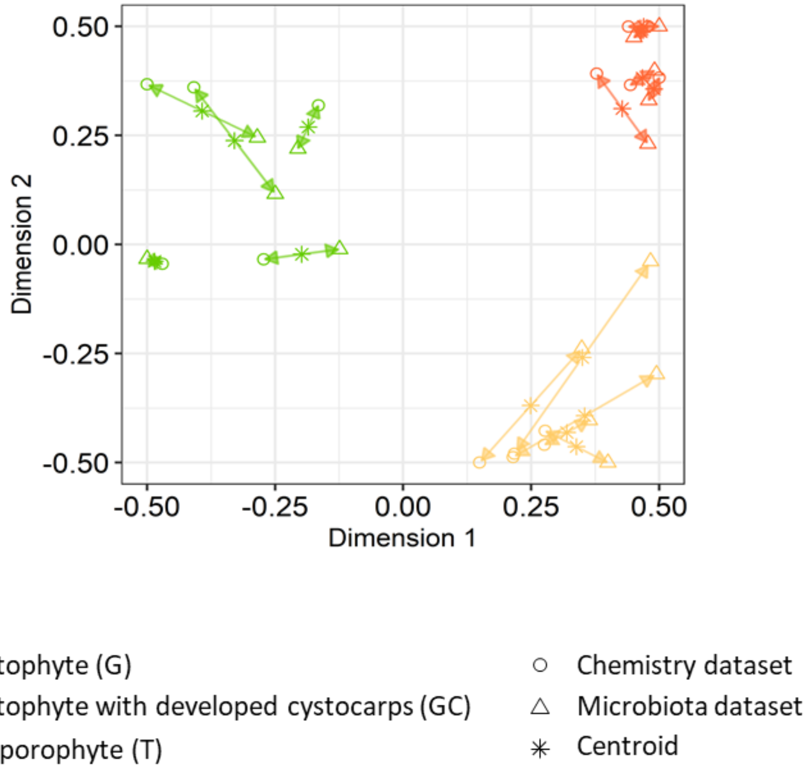

Figure S15: Correlations between the first dimension (A) and the second dimension (B) of each dataset (Chem= Chemistry; Metabarc= Metabarcoding) for the two PLS models. Codes "C" represents samples of the gametophyte with developed cystocarps, "G" the gametophyte samples and "T" the tetrasporophyte samples.

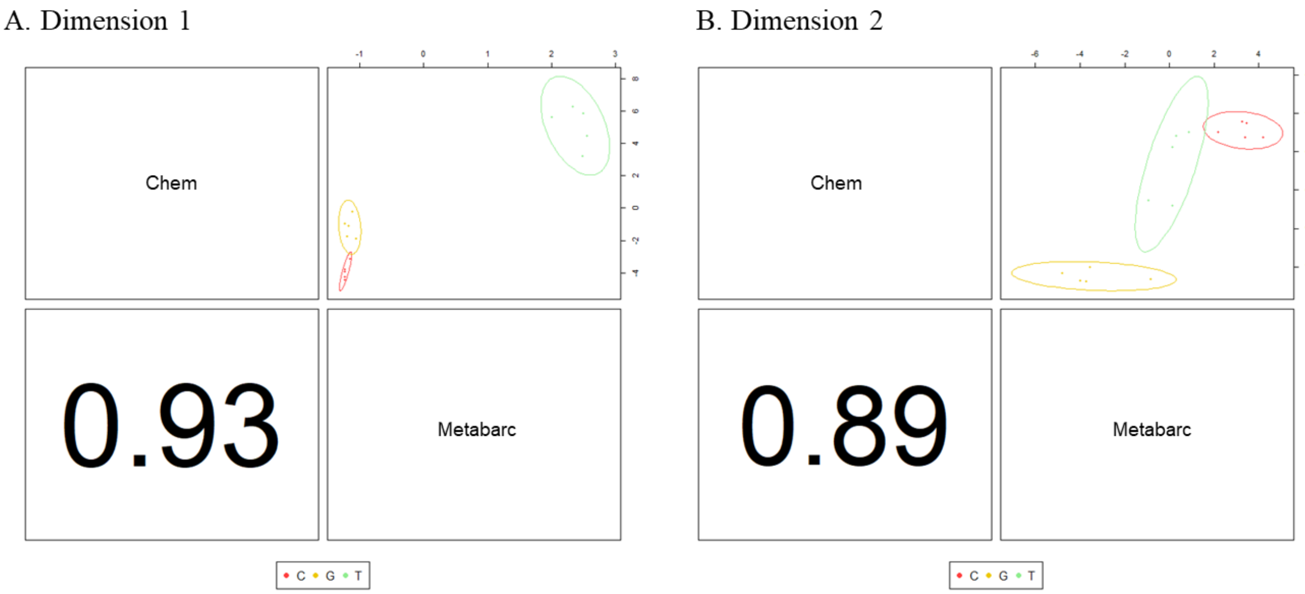

Figure S16: Heatmap of significant ASV and metabolites of the three phases of *A. armata* ("GC" represents samples of the gametophyte with developed cystocarps, "G" the gametophyte samples and "T" the tetrasporophyte samples)

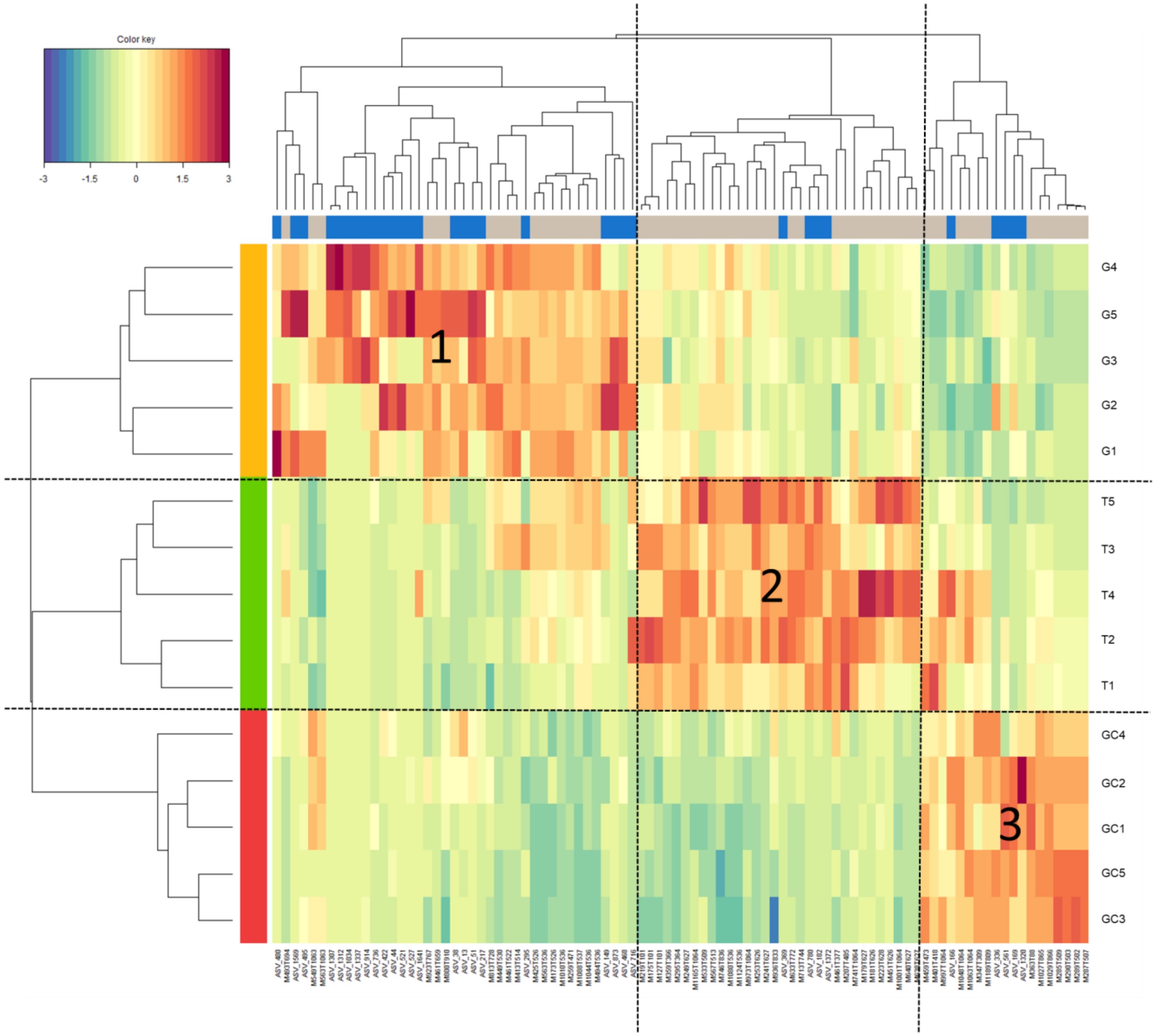

Supplement: Supplementary file 1 [file marinedrugs-21-00363-s001.zip › Supplementary Materials.pdf]
